# Equipoise lost in pancreatic cancer: reimagining crossover for promising cancer therapies

**DOI:** 10.1093/oncolo/oyag347

**Published:** 2026-08-28

**Authors:** Kruti B Vora, Susan E Bates

**Affiliations:** Division of Hematology/Oncology, Columbia University Irving Medical Center, New York, NY 10032, United States; Division of Hematology/Oncology, Columbia University Irving Medical Center, New York, NY 10032, United States; Herbert Irving Comprehensive Cancer Center, Columbia University Irving Medical Center, New York, NY 10032, United States; James J. Peters Bronx VA Medical Center, New York, NY 10468, United States

In diseases with limited prognosis, such as metastatic pancreatic ductal adenocarcinoma (PDAC), time is precious. Median overall survival (OS) from the start of first-line treatment is typically just over 15 months, so any novel therapy that can extend life by even a few months is seen as revolutionary by doctors and patients alike. When such a treatment presents itself through experimental data, the scientific community faces the challenging task of proving the true efficacy and safety of the treatment while also making it available to patients as soon as possible.

Novel treatments approved by the U.S. Food and Drug Administration (FDA) are often based on high-quality randomized controlled trials showing that improvement of a clinical trial endpoint such as OS or a surrogate such as progression-free survival (PFS) is due to the experimental agent rather than an unanticipated bias. The delicate balance of a randomized trial rests on the principle of equipoise, a “state of genuine uncertainty” about the possibly superior treatment arm, which justifies keeping a clinical trial open and exposing patients to either the experimental or control treatments.^1^ The case of daraxonrasib, the novel KRAS inhibitor that reported a doubling of the median overall survival for patients with previously treated metastatic PDAC, points to exactly the role of equipoise.^2^

## RASolute-302: a study in equipoise lost

The current standard of care treatment of metastatic PDAC is based on a chemotherapy backbone of either 5-fluorouracil, oxaliplatin, irinotecan, leucovorin (FOLFIRINOX), or gemcitabine and nab-paclitaxel (Gem/Abraxane).^3^^,^^4^ For years, clinicians and scientists have tried to expand the options beyond these chemotherapies, hoping to extend patient survival. Novel therapeutic agents such as pegvorhylauronidase alfa, napabucasin, devimistat, ibrutinib, and others reached phase III trials but failed to improve OS or PFS.^5–9^ These studies show why randomization is considered important—in each case, the novel therapy was thought to be better, and in each case it was not.

Enter daraxonrasib, a novel molecular glue binding cyclophilin A to inhibit activated RAS(ON) complexes, offering a promising way to block downstream activation of the RAS/MAPK pathway for pan-RAS mutations. Revolution Medicines first began targeted laboratory studies of daraxonrasib (RMC-6236) following its October 2018 acquisition of Warp Drive Bio. Following intense structure-guided laboratory design and optimization, RMC-6236 emerged as the leading candidate. The first-in-human trial, RMC-6236-001 (NCT05379985), enrolled patients beginning June 22, 2022.^10^ As early as February 27, 2023, a press release noted that daraxonrasib could shrink pancreatic cancers, accepted by many as an important surrogate in PDAC despite being debated in other tumor types.^11^ The first publicly presented clinical results for daraxonrasib came in October 2023, roughly 16 months after the first patient was dosed.^12^ Preliminary PFS and OS data were reported in October 2024.^13^ These exciting findings led to several daraxonrasib trials including the pivotal trial in second line (RASolute-302), a first-line trial in combination with chemotherapy (RASolute-303), and a trial in the adjuvant/maintenance setting (RASolute-304). At some point, equipoise was lost.

The RASolute-302 pivotal trial did not allow crossover, so patients randomized to the control arm were not able to benefit from the groundbreaking discovery. At institutions where RASolute-302 was open, there was heartbreak for patients who were randomized to the control arm. These patients’ contribution to the field and to patients currently struggling with pancreatic cancer cannot be overstated. Like others, one of our patients rapidly lost heart after randomizing to chemotherapy and opted for hospice instead of continued treatment. After the RASolute-302 results were announced, his wife noted that he would have been glad to know that he contributed to the findings. The field is grateful to every patient who enrolled in the study, but candidly, noble sacrifice in the face of death is a literary archetype that should be minimized in the ranks of oncology drug development, particularly in PDAC, where on average 150 patients die in the US each day. There must be a better way.

RASolute-302, the pivotal second-line trial that will anchor the drug’s upcoming approval, was designed as a phase III, international, open-label randomized controlled trial for patients with previously treated metastatic PDAC to either daraxonrasib 300 mg daily or standard of care second-line chemotherapy. As noted, crossover was not allowed, and OS was a dual primary endpoint with PFS. Five hundred patients were accrued rapidly between October 16, 2024, and November 7, 2025, with the first interim analysis planned after 166 events in patients with tumors harboring KRAS G12 mutations. At the initial data cutoff (February 10, 2026), the median time of follow-up was a short 8.5 months. The results, presented at the plenary session of the 2026 American Society of Clinical Oncology (ASCO) Annual Meeting, received a standing ovation. The median OS of patients on daraxonrasib was 13.2 months compared to 6.7 months with standard chemotherapy.^2^ Daraxonrasib was accepted for FDA regulatory review for previously treated metastatic pancreatic cancer on July 22, 2026, and was approved on August 26, 2026.

There remains no doubt that daraxonrasib is active against PDAC. However, a closer look at the results of RASolute-302 demonstrates equipoise was lost early on. Thirty-eight (15.1%) patients randomized to the chemotherapy arm were never treated on the study. Undoubtedly most were patients who hoped they would randomize to daraxonrasib, an oral medication with great promise, and avoid another chemotherapy they felt would not work and only bring further toxicity. Another 69 patients (27.4%) were either withdrawn by the investigator (29), withdrew voluntarily (26), or discontinued for an adverse event (14) before scoring disease progression, explaining a chemotherapy duration of treatment of only 1.5-3.2 months depending on the regimen, less than many patients in the real world receive in the second-line setting. Thus, a total of 107 (42.5%) control arm patients were either not treated or insufficiently treated and were censored for the PFS endpoint, a censoring rate that renders any Kaplan–Meier difficult to interpret.^14^ The OS Kaplan–Meier curve, less vulnerable to censoring, shows an early split of the daraxonrasib curve at the one-month mark with consistent improvement over chemotherapy. However, the median follow-up was only 8.5 months. In standard Kaplan–Meier statistics, when the calculated median OS vastly exceeds the median follow-up, its reliability is diminished. We should view the 13.2-month OS as an encouraging, early signal rather than a finalized fact. As the trial progresses and the median follow-up reaches 15 to 20 months, the median OS will “settle” into its true, stable value, and we will better understand the contribution of daraxonrasib to the management of pancreatic cancer.

## Historical adjustments in trial conduct for equipoise

There is historical precedent for studies to be stopped or amended early when equipoise is lost; either the experimental arm outperforms the control or vice versa. For example, in CheckMate-066, patients with metastatic melanoma were randomized to receive nivolumab (experimental) or dacarbazine.^15^ An unplanned early interim analysis showed a trend toward an OS benefit with nivolumab, so the study’s monitoring committee recommended unblinding the study and giving patients in the control arm access to nivolumab.

The concern has been that stopping trials early may result in a large effect size and overemphasis of the true benefit of an experimental intervention.^16^^,^^17^ However, one analysis of 18 National Cancer Institute Cooperative Group trials stopped early for positive results showed that 17 of the 18 trials had a comparable or only modestly smaller effect size at final analysis compared to the early stopping point.^18^ The ability to stop a trial early with confidence relies on well-designed interim monitoring plans. The first interim analysis for RASolute-302, which was conducted 16 months after the trial opened, after all patients were enrolled, raises the question of whether efficacy could have been assessed earlier. Earlier interim monitoring may lead to earlier access for promising novel cancer treatments.

## Expediting drug approval pathways

As more novel cancer therapies enter development, scientific and regulatory communities must distinguish true clinical benefit from promising early signals that don’t ultimately result in meaningful patient outcomes. The traditional model of drug development is linear; a new therapy is studied in preclinical laboratory work, then phase I, phase II, and phase III trials are conducted prior to FDA/European Medicines Agency (EMA) review, approval, and eventual access. This model is designed to ensure patient safety and is useful when there is continued uncertainty about a treatment. However, the process is lengthy: only about 1 in 5 drugs that are tested in phase I trials end up with FDA approval, and it typically takes over 8 years for a drug to advance from preclinical models to FDA approval.^19^

When more urgent approvals are warranted, the regulatory approval process can and should be expedited, and additional efforts are being made to conduct FDA reviews in parallel with clinical trials via Real-Time Oncology Review (RTOR) and Real-Time Clinical Trials (RTCTs). The current standard for urgent drug approval is accelerated approval, usually granted by using a surrogate or intermediate clinical endpoint to allow for medication access prior to mature OS data. Although disliked by many, accelerated approval can be valuable if the required confirmatory trials are completed.^20^^,^^21^ Olaratumab, a monoclonal antibody granted accelerated approval by the FDA in October 2016 for advanced soft tissue sarcoma, is an excellent example of equipoise lost leading to a false phase II study result. The study was open label, with doxorubicin given in both the experimental arm with olaratumab and as monotherapy in the control arm. Patients in the control arm received half as much doxorubicin as in the experimental arm, so the difference in doxorubicin dose and not olaratumab ultimately drove the results. Its approval was rescinded when a larger blinded phase III confirmatory trial that administered comparable doxorubicin to both arms failed to show an OS benefit.^21^

Although one can argue OS should be the approval endpoint for a disease such as PDAC even in first line, if the study is conducted with crossover, a meaningful response rate and duration of response together with a promising PFS could support approval. Crossover would then allow all who enroll to receive daraxonrasib and additionally assess a maintenance approach that could emerge as superior if it allowed the initial chemotherapy to be given without its dose/schedule impacted by the toxicities of concurrent daraxonrasib, additionally answering the question of whether daraxonrasib first or later is better. Indeed, such a study would succeed even if the arms were comparable by showing an oral targeted therapy was equivalent to intravenous chemotherapy. A pre-planned design with adjustments for crossover will be better than having 37.5% withdraw, having either not been treated or been insufficiently treated and censored for the PFS endpoint as occurred in RASolute-302. Late-phase clinical trials should have mechanisms to accelerate or provide early drug access when a drug has promising success, and the FDA/EMA should make clear to companies that a result possibly confounded by crossover will not prevent drug approval.^22^

Trials are often designed to detect failure, but they should also be built to respond to unexpected success. By instituting earlier interim analyses and prespecified crossover strategies, patients could gain access to promising therapies while preserving the randomized evidence needed to establish efficacy. These approaches recognize that ethical justification for randomization can change as evidence accumulates. For patients with metastatic PDAC, delays of months for treatment access are not trivial. This could represent a substantial portion of a patient’s remaining window for treatment. RASolute-302 therefore highlights a larger challenge in drug development and trial design: clinical equipoise should be assessed dynamically with trial design and regulatory pathways able to adapt when compelling evidence emerges that puts equipoise in peril. The choice should not be between rigorous evidence and timely access, but to reimagine crossover and build a system capable of achieving both.
